# Data-driven modeling of water adsorption isotherms in cocoa beans: Dataset and Python-based Machine Learning tools for multivariate analysis and storage management

**DOI:** 10.1016/j.dib.2026.112616

**Published:** 2026-02-19

**Authors:** Andrés F. Bahamón-Monje, Gentil A. Collazos-Escobar, Nelson Gutiérrez-Guzmán

**Affiliations:** a Centro Surcolombiano de Investigación en Café (CESURCAFÉ), Departamento de Ingeniería Agrícola, Universidad Surcolombiana, Neiva-Huila, 410001, Colombia; b Departamento de Ingeniería Agroindustrial, Facultad de Ingeniería, Universidad Surcolombiana, Neiva, Huila, Colombia; c Grupo de Análisis y Simulación de Procesos Agroalimentarios (ASPA), Instituto Universitario de Ingeniería de Alimentos-FoodUPV, Universitat Politècnica de València, Camí de Vera s/n, Edificio 3F, València, 46022, España

**Keywords:** Hygroscopicity, Water adsorption properties, Moisture stability, Artificial intelligence, Predictive model, Storage optimization, Quality management

## Abstract

This work presents a comprehensive dataset of water adsorption isotherms for dried and roasted cocoa beans (*Theobroma cacao* L.), complemented by a set of Python-based machine learning (ML) tools designed to support data-driven multivariate modeling and storage management. Adsorption isotherms were experimentally determined using the Dynamic Dewpoint Isotherm (DDI) method in the range of water activity (a_w_) of 0.1–0.85 and at temperatures representative of post-harvest storage facilities (25, 30, and 40°C). The water adsorption isotherms provided a detailed analysis of the hygroscopic behavior of dried and roasted cocoa beans, enabling the assessment of moisture-related stability during storage. The dataset includes Excel files containing equilibrium moisture content (X_e_) values, experimental conditions (a_w_, temperature, and type of cocoa beans) and replicate measurements, thereby enabling reproducible and traceable modeling workflows. To complement the experimental data, this work also provides fully documented Python scripts for multivariate mathematical modeling and prediction of water adsorption behavior using ML techniques. The computational ML tools were developed in Python using the Spyder integrated development environment (IDE) provided by the Anaconda distribution, with libraries such as *scikit-learn* and *pandas*. These tools implement automated routines for model calibration and grid search-based hyperparameter optimization of Support Vector Machine (SVM), Random Forest (RF), and Artificial Neural Networks (ANN). These models enable the prediction of X_e_ as a function of a_w_, temperature, and cocoa processing type (dried or roasted), supporting robust multivariate modeling strategies. The integrated dataset and Python workflow together constitute a methodological framework for predicting moisture behavior in cocoa during storage, assessing storage-related risks based on model outputs, and supporting informed decision-making throughout the cocoa supply chain. By enabling reliable estimation of moisture-related storage parameters in cocoa, critical aspects associated with stability, spoilage prevention, and shelf-life determination can be addressed. Consequently, this work provides a valuable reference dataset for researchers, cocoa producers, and industry stakeholders seeking to optimize storage conditions and maintain cocoa quality during post-harvest handling.

Specifications Table

**Table:** 

Subject	Food Science
Specific subject area	Food Science and Technology, Food engineering.
Type of data	Excel files: (initial characterization, water adsorption isotherms) Python scripts (SVM, RF and ANN data-driven modeling routines)
Data collection	Initial characterization (moisture; gravimetrically obtained, mid-infrared spectra; obtained by Attenuated Total Reflectance-Fourier Transform Infrared; ATR-FTIR). Water adsorption isotherms (obtained by DDI method).
Data source location	The experimental data obtained in this work were collected at the Centro Surcolombiano de Investigación en Café (CESURCAFÉ) of the Universidad Surcolombiana in Neiva, Huila, Colombia.
Data accessibility	Repository name: Mendeley Data Data identification number: 10.17632/nwkdf22cn9.2 Direct URL to data: https://data.mendeley.com/datasets/nwkdf22cn9/2
Related research article	None

## 1 Value of the Data

• The dataset provides experimentally determined water adsorption isotherms for dried and roasted cocoa beans under storage-relevant temperature and a_w_ conditions, enabling a quantitative assessment of cocoa hygroscopic behavior during storage.
• This dataset supplies a basis for food scientists, post-harvest technologists, and cocoa processors to quantitatively analyze moisture-related storage behavior and optimize handling strategies. Additionally, quality control professionals, supply chain stakeholders, and regulatory bodies may use the dataset to support evidence-based decision-making aimed at mitigating moisture-driven spoilage risks in cocoa and related agro-food products.
• The high-resolution water adsorption data can be directly used to train, validate, and benchmark ML models for predicting X_e_ and moisture-related storage stability in cocoa beans.
• The analysis of cocoa hygroscopicity at dried and roasted stages enables comparative evaluation of processing effects on water adsorption behavior, supporting improved understanding of roasting-induced changes in cocoa hygroscopic properties.
• The dataset supports the development of data-driven and multivariate modeling approaches applicable to post-harvest storage and processing optimization, contributing to improved quality inspection and risk mitigation throughout the cocoa supply chain.
• The Python-based ML tools provide accessible and reproducible computational workflows that can be directly applied by researchers and industry stakeholders to support decision-making related to moisture control and storage risk assessment. The implemented modeling framework is transferable and can be adapted to other agricultural and food products, extending the applicability of the dataset beyond cocoa.

## 2 Background

Cocoa plays a strategic role in the economies of producing countries and in the global food industry [1], owing to its distinctive sensory properties and its widespread use as a key raw material in chocolate, beverages, confectionery, bakery products, and functional foods [2]. Beyond its economic importance, cocoa quality is a critical determinant of market value and consumer acceptance, making post-harvest handling and storage decisive stages within the cocoa supply chain. Among the factors affecting quality during storage, moisture adsorption is one of the most influential, due to the strongly hygroscopic nature of cocoa beans and their sensitivity to fluctuations in environmental relative humidity and temperature [3].

Inadequate moisture control during storage can trigger a cascade of undesirable phenomena, including physicochemical degradation, lipid oxidation, flavor deterioration, caking, and microbial growth, ultimately leading to economic losses and reduced shelf life [4]. Consequently, a precise understanding of the moisture-a_w_ relationship is essential for defining safe storage thresholds, predicting quality stability, and implementing effective risk management strategies. Water adsorption isotherms provide the fundamental framework for describing these interactions, enabling the quantification of X_e_ as a function of a_w_ and temperature under storage-relevant conditions [5].

The present dataset was generated using the DDI method, a high-resolution and time-efficient technique that offers significant advantages over conventional static methods based on saturated salt solutions [6]. The DDI approach allows rapid acquisition of reliable sorption data while minimizing structural alterations and microbial interference during measurements [7]. These characteristics are particularly valuable for generating datasets intended not only for descriptive analysis but also for advanced computational modeling [8]. The resulting water adsorption data enable the estimation of key water adsorption and thermodynamic properties that are directly linked to moisture-driven stability and storage behavior in cocoa beans [9].

In addition to its relevance for classical sorption modeling [10], this dataset is specifically designed to support data-driven and ML-based approaches for quality assessment and storage management. Traditional theoretical and empirical models (e.g., BET, GAB, Oswin, Halsey, and Henderson) often rely on simplified physically based assumptions, which limit their ability to capture complex, non-linear trends and to handle a large number of input variables and data within multivariate modeling frameworks [11]. Conversely, data-driven modeling approaches based on ML techniques such as SVM, RF, and ANN are capable of learning multivariate, nonlinear relationships directly from experimental data, offering a flexible and robust alternative for calibrating predictive models across diverse conditions [12].

The analysis of dried and roasted cocoa beans substantially enhances the scientific value of this dataset by enabling the development of robust predictive models capable of simultaneously estimating cocoa X_e_ while accounting for the post-harvest processing stage. Additionally, it supports a comparative assessment of cocoa hygroscopic behavior, allowing the quantification of roasting-induced effects on water adsorption. Roasting induces pronounced structural, physicochemical, and compositional modifications that alter water-solid interactions within the cocoa matrix; these process-driven changes can be effectively captured, parameterized, and modeled using data-driven approaches.

By integrating high-resolution adsorption data with ML tools implemented in open-source software (such as Python), the dataset facilitates the development of predictive models capable of estimating X_e_ as a function of a_w_, temperature, and cocoa processing state. The ML modeling framework supported by this dataset establishes a foundation for the development of decision-support systems for cocoa storage management [13]. When coupled with real-time sensor data from storage environments (temperature and relative humidity sensors), ML-trained/validated models can be used to make accurate real-time predictions of moisture and quality-related parameters, thereby enabling quality control strategies. These digital tools contribute to the design of intelligent storage systems, enhanced quality monitoring, and model-based decision-making throughout the cocoa supply chain.

## 3 Data Description

The experimental dataset was compiled in two Excel files and three Python scripts, according to the initial characterization of dried and roasted cocoa beans, their water adsorption isotherms, and the application of data-driven ML tools for multivariate modeling of water adsorption isotherms, simultaneously considering the influence of a_w_, temperature, and cocoa type in the description of X_e_.

**Fig. 1:**
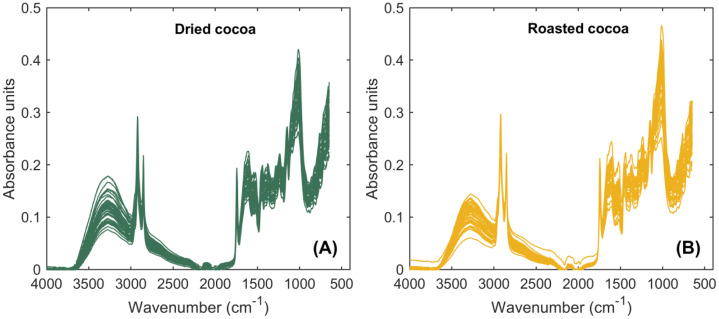
Fourier Transform Infrared (FTIR) spectra of cocoa beans: dried cocoa (A) and roasted cocoa (B). Mid-infrared spectra were obtained in the wavenumber range of 4000–500 cm^–1^ plotted as a function of absorbance units.

**Initial_characterization_Cocoa:** This file summarizes initial moisture and spectra-related data of dried and roasted cocoa beans. Sample identification (n=15; see the EXPERIMENTAL DESIGN, MATERIALS AND METHODS section) is reported in the first column, followed by the replicate number (n = 3) in the second column. The third and fourth columns correspond to the X_e_, expressed on a wet basis ( % w.b.) and dry basis ( % d.b.), respectively. The fifth column reports the a_w_ measured for each sample and replicate. Regarding the spectral properties of cocoa (Fig. 1), columns 6 to 905 contain the mid-infrared spectral data. Each column corresponds to a specific wavenumber (cm⁻¹), which remains constant across all samples and replicates. The wavenumbers are reported in the header row and span the mid-infrared region. Additionally, this Excel file is divided into two sheets, corresponding to dried and roasted cocoa beans, respectively, while maintaining an identical data structure to facilitate comparative analysis and multivariate modeling.

**Fig. 2:**
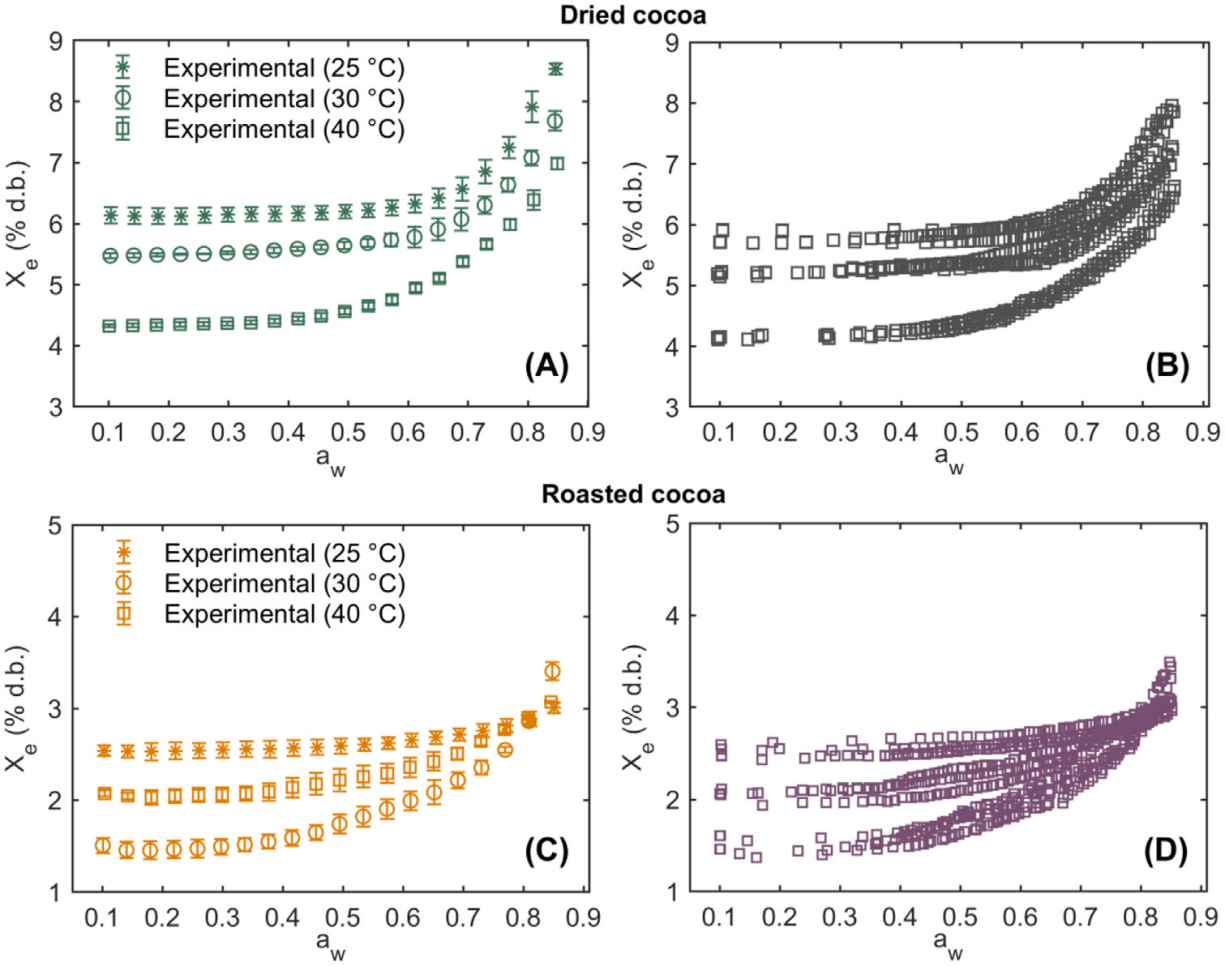
Experimental water adsorption isotherms of dried and roasted cocoa beans as a function of water activity (a_w_) at temperatures of 25, 30, and 45°C. Isotherms are presented as mean ± standard deviation (A) and individual replicates (B) for dried cocoa beans, and as mean ± standard deviation (C) and individual replicates (D) for roasted cocoa beans. Equilibrium moisture content (X_e_) values are expressed on a dry basis ( % d.b.).

**WaterAdsorptionIsotherms_DriedRoasted_Cocoa:** This Excel file compiles the water adsorption isotherm data for dried and roasted cocoa beans. The dataset includes a_w_ values ranging from 0.10 to 0.85, three temperature levels (25, 30, and 40°C), and the X_e_ expressed as a percentage on both wet ( % w.b.) and dry ( % d.b.) bases. The first column contains a dummy variable indicating the cocoa processing state (0 = roasted, 1 = dried). The second column identifies the experimental replicates (n=5), while columns four to six report the corresponding a_w_ values and X_e_. Fig. 2 presents the water adsorption isotherms of dried and roasted cocoa beans at different experimental temperatures over an a_w_ range of 0.10–0.85. The isotherms are displayed as mean values with corresponding standard deviations, calculated by considering all experimental replicates (n=5) for each temperature and cocoa type. Additionally, the figure shows the individual replicates performed for each experimental condition. The water adsorption isotherms for both dried (Fig. 2, Fig. 2) and roasted (Fig. 2, Fig. 2) cocoa beans exhibit a characteristic J-shaped curve, corresponding to Type III isotherms according to the Brunauer-Emmett-Teller (BET) classification [14]. This behavior is commonly observed in food matrices rich in sugars and other soluble compounds, such as baked or roasted products [15,16].

**SupportVectorMachineModeling_WaterAdsorptionIsotherms.py:** This Python script was designed to perform SVM regression modeling of experimental water adsorption isotherm data obtained from dried and roasted cocoa beans. The main goal of the script was to develop, optimize, and assess an SVM-based predictive model for predicting the X_e_ of cocoa beans as a function of a_w_, temperature, and cocoa processing type.

The script begins by importing the required scientific computing and ML libraries, including *pandas, scikit-learn*, and *matplotlib*. Experimental sorption isotherm data are then loaded from an Excel file (**WaterAdsorptionIsotherms_DriedRoasted_Cocoa.xlsx**). From this dataset, the cocoa type (encoded as a dummy variable), temperature, a_w_, and X_e_ are defined. Cocoa type is incorporated as an input feature to enable a multivariate modeling approach, allowing the SVM to learn differences between dried and roasted cocoa beans within a combined framework. The input matrix consists of cocoa type, a_w_, and temperature, while X_e_ serves as the response variable. The dataset is randomly split into training (75 %) and validation (25 %) subsets, using a fixed random seed to ensure reproducibility. Prior to model training, all input features are standardized using z-score normalization, a crucial step for SVM regression to ensure numerical stability and balanced feature influence, particularly when kernel-based methods are employed.

SVM regression is implemented using the SVR class from *scikit-learn*. Model optimization was carried out through an exhaustive grid search strategy using the *GridSearchCV* function, combined with 5-fold cross-validation on the training dataset. The hyperparameter search space includes multiple values of the regularization parameter (C = 0.1, 1, 10, 100), different kernel functions (linear, radial basis function, and polynomial), and alternative gamma settings (scale and auto). Model performance during optimization was assessed using the mean squared error (MSE, Eq. 1), and the hyperparameter combination yielding the lowest cross-validated error was selected as the optimal model. Once the optimal SVM configuration was identified, predictions were generated for both training and validation datasets. Model performance was assessed using different goodness-of-fit metrics, including mean absolute error (MAE, Eq. 2), mean squared logarithmic error (MSLE, Eq. 3), median absolute error (MedAE, Eq. 4), maximum error (ME, Eq. 5), coefficient of determination (R^2^, Eq. 6), and explained variance (VAR, Eq. 7–9). These figures-of-merit provide complementary insights into prediction accuracy, error distribution, and variance explained by the model. All performance indicators for training and validation datasets were compiled into a summary table and enable users to export all the results as an Excel file (*Support Vector Machine modeling performance.xlsx*).

(1) $$\text{MSE}\;\left(\text{squared}\;\text{RU}\right)=\frac{\sum_{i=1}^{N}{\left(Y_{\text{exp}}-\;Y_{\text{pred}}\right)}^{2}}{N}$$

(2) $$\text{MAE}\;\left(\text{RU}\right)\;=\frac{1}{n}\sum_{i=1}^{n}\left|Y_{\text{exp}}-Y_{\text{pred}}\right|$$

(3) $$\text{MSLE}\;\left(\log\text{squared}\;\text{RU}\right)=\frac{\sum_{i=1}^{N}{\left(\text{log}\left(1+Y_{\text{exp}}\right)-\;\text{log}\left(1+Y_{\text{pred}}\right)\right)}^{2}}{N}$$

(4) $$\text{MedAE}\;\left(\text{RU}\right)\;=\text{Median}\left(\left|Y_{\text{exp}}-Y_{\text{pred}}\right|\right)$$

(5) $$\text{ME}\;\left(\text{RU}\right)\;=\text{Max}\left(Y_{\text{exp}}-Y_{\text{pred}}\right)$$

(6) $$R^{2}\left(\%\right)=100-\frac{\sum_{i=1}^{n}{\left(Y_{\exp\;}-Y_{\text{pred}}\right)}^{2}}{\sum_{i=1}^{n}{\left(\bar{Y_{\exp\;}}-\;Y_{\text{epred}}\right)}^{2}}$$

(7) $$\text{VAR}\left(\%\right)=100-\left(1-\frac{S_{\text{yx}}^{2}}{S_{\text{yx}}^{2}}\right)$$

(8) $$S_{y}=\sqrt{\frac{1}{n-1}\sum_{i=1}^{n}{\left(Y_{\text{exp}}-\;Y_{\text{pred}}\right)}^{2}}$$

(9) $$S_{\text{yx}}=\sqrt{\frac{1}{n-m}\sum_{i=1}^{n}{\left(Y_{\text{exp}}-\;Y_{\text{pred}}\right)}^{2}}$$

Where Y_exp_ and Y_pred_ are the experimental and predicted X_e_ by the ML models, RU is the units of response variable (in this case %d.b.), n is the number of experimental observations in the corresponding dataset and m is the number of model’s parameters.

Given the data-driven nature of modeling water adsorption isotherms, there is no single ML model or hyperparameter configuration that can adequately describe all agricultural and food products due to differences in composition, structure, and hygroscopic behavior. Therefore, the ML models selected in this work were chosen based on their complementary learning capabilities, robustness for nonlinear regression, and widespread use in food science and physicochemical modeling applications.

Specifically, the selected techniques represent a balance between model interpretability, predictive performance, and computational efficiency, making them well-suited for benchmarking X_e_ prediction from experimental adsorption data. To avoid model-specific bias and to enhance adaptability, the Python script developed in this study enables users to easily expand or modify the hyperparameter search space, including the incorporation of additional kernel functions and regularization parameters. This flexibility facilitates deeper exploration of model complexity and supports improved generalization performance beyond the configurations evaluated in this work.

Additionally, the script generates several graphical outputs to facilitate the visual interpretation of model performance. These include: (i) experimental vs. predicted water adsorption isotherms as a function of a_w_ for both training (75 %) and validation (25 %) datasets, and (ii) fitting results plots comparing the agreement between experimental and predicted X_e_, including a 1:1 reference line to evaluate the visual accuracy of prediction and any dispersion mismatches between Y_exp_ and Y_pred_. As a result, this script allows users to save the SVM’s performance figure in high-resolution TIFF format (*SupportVectorMachineResults.tif*). This Python-based SVM modeling framework provides a robust and flexible approach for capturing the nonlinear relationships governing water adsorption behavior in cocoa beans while enabling systematic hyperparameter optimization, rigorous model validation, and comprehensive performance analysis.

**RandomForestModeling_WaterAdsorptionIsotherms.py:** This Python script implements an RF regression modeling framework to predict the X_e_ dried and roasted cocoa beans based on experimental water adsorption isotherm data. The model leverages key explanatory variables (a_w_, temperature, and cocoa type), to describe nonlinear relationships governing water adsorption behavior in cocoa matrices. The script begins by importing the necessary Python libraries for data processing, ML, and visualization, including *pandas, scikit-learn*, and *matplotlib*. Experimental data was loaded from the Excel file *WaterAdsorptionIsotherms_DriedRoasted_Cocoa.xlsx*, from which cocoa type (encoded as a numeric dummy variable), temperature, a_w_, and X_e_ were extracted. A multivariate modeling strategy was adopted by including cocoa type as an input feature, enabling the RF model to simultaneously learn patterns associated with both dried and roasted cocoa beans. The predictor matrix consists of cocoa type, a_w_, and temperature, while X_e_ was used as the response variable. The whole dataset was randomly partitioned into training (75 %) and validation (25 %) subsets, using a fixed random seed to ensure the reproducibility of the results.

RF regression was performed using the *RandomForestRegressor* class from *scikit-learn*. Model optimization was conducted via an exhaustive grid search combined with 5-fold cross-validation (*GridSearchCV*), with the objective of minimizing the MSE on the training data. A comprehensive hyperparameter space was explored, including the number of decision trees (n_estimators = 1 to 10.000), maximum tree depth (None, 5, 10, 15, and 20), minimum number of samples required for node splitting and leaf nodes, and different strategies for feature selection at each split (square root, logarithmic, or all features). This systematic search allows for robust identification of an optimal model configuration while balancing model complexity and predictive accuracy. After selecting the optimal hyperparameter combination, the best-performing RF model was used to generate predictions for both the training and validation datasets. Model performance was quantitatively assessed using several goodness-of-fit metrics, including MAE, MSE, MSLE, MedAE, ME, R^2^, and VAR. These metrics provided a comprehensive assessment of prediction accuracy, error scattering, and variance explanation. The resulting performance indicators for the training and validation datasets were compiled into a summary table and exported to an Excel file (*Random Forest modeling performance.xlsx*).

The Python framework presented for training and validating a RF, enables users to systematically extend the hyperparameter grid, including the more number of trees, tree depth, and feature selection strategies. This tool supports enhanced exploration of ensemble complexity and optimization across different datasets (modeling other food products) and experimental conditions.

To support visual aid of model performance, this script generates a set of figures. These include comparisons between experimental and predicted water adsorption isotherms as a function of a_w_ for both training and validation datasets, as well as parity plots illustrating the agreement between experimental and predicted X_e_ values. All plots were consistently formatted and combined into a single multi-panel figure, which is saved in high-resolution TIFF format (*RandomForestResults.tif*), suitable for scientific dissemination. This RF regression framework provides a powerful, nonparametric modeling approach capable of capturing complex nonlinear interactions between storage related variables (a_w_ and temperature) and type of cocoa, offering a robust alternative to kernel-based ML techniques for predicting water adsorption behavior in cocoa beans.

**ArtificialNeuralNetworksModeling_WaterAdsorptionIsotherms.py:** This Python script implements an ANN regression framework to model and predict the X_e_ of dried and roasted cocoa beans using experimental water adsorption isotherm data. The ANN approach was used to model complex, nonlinear relationships between X_e_ and a_w_, temperature, and cocoa processing type. Experimental data were directly loaded from the Excel file (previously described), from which cocoa type (encoded as a numeric dummy variable), temperature, a_w_, and X_e_ were obtained. A multivariate modeling strategy was adopted by incorporating cocoa type as an input feature, enabling the ANN to simultaneously learn from the water adsorption behavior of both dried and roasted cocoa beans. The predictor matrix was constructed using cocoa type, a_w_, and temperature, while X_e_ served as the response variable. The dataset was randomly divided into training (75 %) and validation (25 %) datasets, with a fixed random seed to ensure reproducibility. Prior to model training, all input features were standardized using z-score normalization, which was essential for ANN training stability and efficient convergence, particularly when gradient-based optimization algorithms were used.

ANN regression was implemented using the *MLPRegressor* class from *scikit-learn*. Model optimization was achieved via an exhaustive grid search combined with 5-fold cross-validation (*GridSearchCV*), with the objective of minimizing the MSE on the training dataset. The hyperparameter search space includes different network architectures (single and double hidden layers with varying neuron counts), activation functions (ReLU and Tanh), optimization solvers (Adam and stochastic gradient descent), and L2 regularization strengths (α). The maximum number of training iterations is set to 2000 to ensure convergence of the learning algorithm. Following hyperparameter optimization, the best-performing ANN model was selected and used to generate predictions for both training and validation datasets. Model performance was quantitatively evaluated using a comprehensive set of goodness-of-fit metrics, including MAE, MSE, MSLE, MedAE, ME, R^2^ and VAR. These metrics assess prediction accuracy, robustness, and variance explanation. Performance results are summarized in a table and exported to an Excel file (*Artificial Neural Networks modeling performance.xlsx*). The ANN regression framework offers a flexible and powerful data-driven modeling approach for describing water adsorption isotherms in cocoa beans, complementing both SVM and RF ML tools to effectively model the water adsorption process in cocoa beans. Additionally, considering the inherently data-driven and architecture-dependent nature of ANN, this Python script allows users to modify the network structures and training hyperparameters, such as hidden layer configurations, activation functions, and regularization terms. This capability facilitates further optimization of network complexity and predictive performance beyond the architectures investigated in this work.

**Fig. 3:**
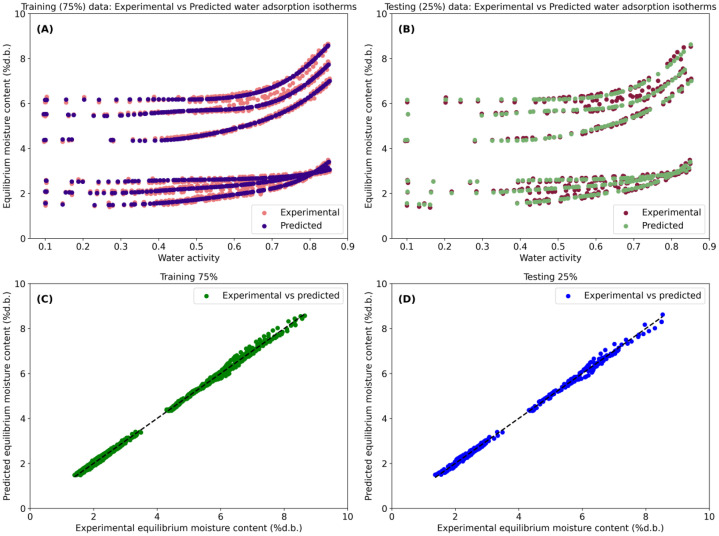
Fitting performance of the Support Vector Machine (SVM) model for predicting equilibrium moisture content (X_e_, % d.b.) as a function of water activity (a_w_), temperature, and cocoa type (dried and roasted beans). Experimental and SVM-predicted water adsorption isotherms for the training (A) and validation (B) datasets and agreement between experimental and predicted X_e_ for training (C) and validation (D).

**Fig. 4:**
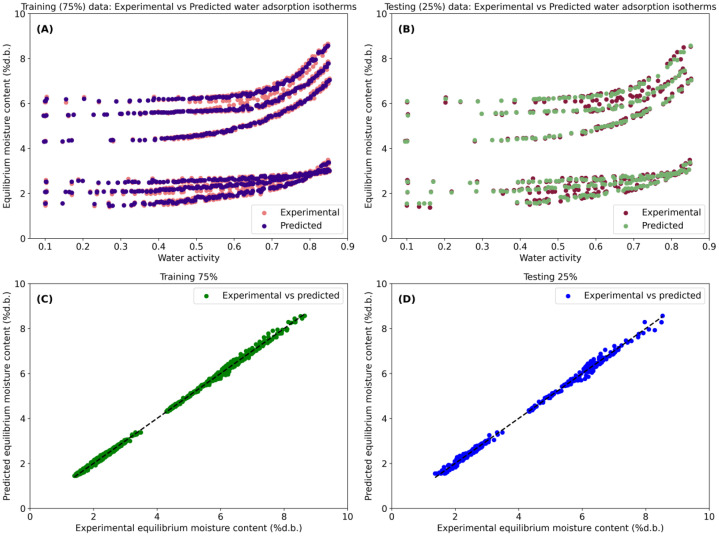
Fitting performance of the Random Forest (RF) model for predicting equilibrium moisture content (X_e_, % d.b.) as a function of water activity (a_w_), temperature, and cocoa type (dried and roasted beans). Experimental and RF-predicted water adsorption isotherms for the training (A) and validation (B) datasets and agreement between experimental and predicted X_e_ for training (C) and validation (D).

**Fig. 5:**
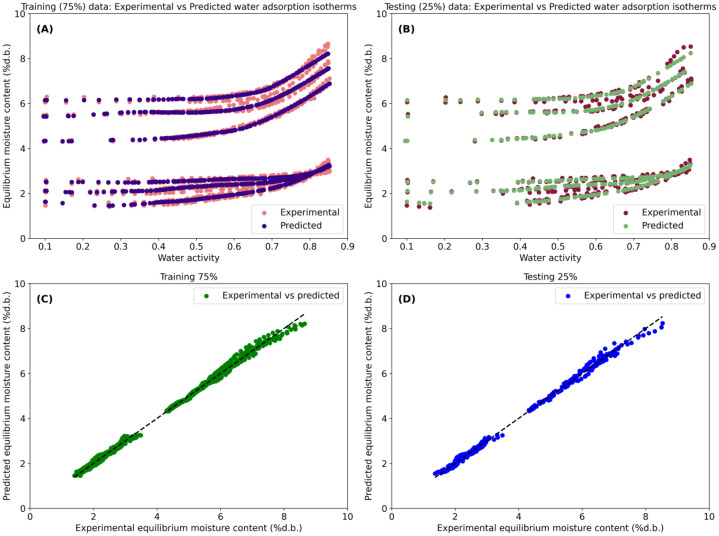
Fitting performance of the Artificial Neural Networks (ANN) model for predicting equilibrium moisture content (X_e_, % d.b.) as a function of water activity (a_w_), temperature, and cocoa type (dried and roasted beans). Experimental and ANN-predicted water adsorption isotherms for the training (A) and validation (B) datasets and agreement between experimental and predicted X_e_ for training (C) and validation (D).

The SVM model (Fig. 3) exhibited excellent predictive performance (R^2^>99 %; Table 1) in describing X_e_ across the full range of a_w_. The predicted isotherms closely matched the experimental data for both the training (Fig. 3A) and validation (Fig. 3B) datasets. The parity plots (Fig. 3, Fig. 3) further demonstrated strong agreement between experimental and predicted X_e_ values for the training and validation datasets, respectively. These results highlight the ability of kernel-based SVM regression to effectively capture the nonlinear relationships between storage-related variables (a_w_ and temperature), cocoa type, and X_e_. Similarly, the RF model fitting results (Fig. 4) showed excellent agreement between experimental and predicted X_e_ values for both the training and validation datasets (R^2^>99 %; Table 2). In addition, the ANN model (Fig. 5) also demonstrated high predictive performance in fitting the experimental data (R^2^>99 %; Table 3).

**Table 1:** Model fitting performance of the grid search-optimized Support Vector Machine (SVM). Results are reported separately for the training (75 %) and validation (25 %) datasets.

Goodness of fit	Training (75 %)	Validation (25 %)
MAE (RU)	0.069	0.073
MSE (squared RU)	6.916 × 10^–3^	8.107 × 10^–3^
MSLE (log squared RU)	3.451 × 10^–4^	3.562 × 10^–4^
MedAE (RU)	0.068	0.070
ME (RU)	0.312	0.318
R^2^ ( %)	99.812	99.778
VAR ( %)	99.811	99.776

MAE (mean absolute error), RU (units of response variable), MSE (mean squared error), MSLE (mean squared logarithmic error), MedAE (median absolute error), ME (maximum error), R^2^ (coefficient of determination) and VAR (explained variance)

**Table 2:** Model fitting performance of the grid search-optimized Random Forest (RF). Results are reported separately for the training (75 %) and validation (25 %) datasets.

Goodness of fit	Training (75 %)	Validation (25 %)
MAE (RU)	0.041	0.069
MSE (squared RU)	3.255 × 10^–3^	9.045 × 10^–3^
MSLE (log squared RU)	1.511 × 10^–4^	4.351 × 10^–4^
MedAE (RU)	0.031	0.048
ME (RU)	0.275	0.355
R^2^ ( %)	99.910	99.751
VAR ( %)	99.910	99.751

MAE (mean absolute error), RU (units of response variable), MSE (mean squared error), MSLE (mean squared logarithmic error), MedAE (median absolute error), ME (maximum error), R^2^ (coefficient of determination) and VAR (explained variance)

**Table 3:** Model fitting performance of the grid search-optimized Artificial Neural Networks (ANN). Results are reported separately for the training (75 %) and validation (25 %) datasets.

Goodness of fit	Training (75 %)	Validation (25 %)
MAE (RU)	0.072	0.076
MSE (squared RU)	9.181 × 10^–3^	0.011
MSLE (log squared RU)	3.981 × 10^–4^	4.591 × 10^–4^
MedAE (RU)	0.055	0.058
ME (RU)	0.441	0.442
R^2^ ( %)	99.747	99.702
VAR ( %)	99.748	99.702

MAE (mean absolute error), RU (units of response variable), MSE (mean squared error), MSLE (mean squared logarithmic error), MedAE (median absolute error), ME (maximum error), R^2^ (coefficient of determination) and VAR (explained variance)

Although the SVM, RF, and ANN models all demonstrated excellent predictive performance (R^2^> 99 % for both training and validation datasets), a comparative analysis of the goodness-of-fit metrics indicates that the RF model provides the best overall performance. The RF model consistently exhibited the lowest error metrics, particularly in terms of MAE, MSE, MSLE, and MedAE, for both the training (MAE = 0.041 RU; MSE = 3.255 × 10^–3^) and validation datasets (MAE = 0.069 RU; MSE 9.045 × 10^–3^), while maintaining very high coefficients of determination (R^2^ > 99.75 %). In comparison, the SVM model showed slightly higher error values, whereas the ANN model presented the largest maximum errors (ME= 0.441 RU), indicating increased sensitivity to data variability. Consequently, when predictive accuracy, error minimization, and robustness across datasets are jointly considered, the RF model can be regarded as the most accurate and reliable approach for predicting X_e_ in this study, with SVM and ANN remaining strong complementary alternatives.

Regarding goodness-of-fit performance metrics, the R^2^ and VAR provided the most consistent and informative indicators for comparative model evaluation, with values exceeding 99.7 % across all models and datasets. Nevertheless, given the similarly high R^2^ values obtained for all ML models, error-based metrics such as MAE, MSE, and MedAE were required to enable a more discriminative assessment of model performance, particularly with respect to generalization capability. Among these indicators, MAE and MSE proved especially relevant for evaluating prediction accuracy and stability between training and validation datasets, whereas MSLE served to verify the absence of systematic bias at low X_e_ values. Consequently, the combined analysis of variance-based and error-based metrics substantiates the robustness of both the dataset and the implemented modeling framework.

All of these machine learning tools can be considered effective for predicting X_e_ and defining appropriate moisture content levels that ensure stability during the storage of dehydrated food products. In storage facilities, these models can be integrated into monitoring systems to support real-time or predictive control of moisture content in stored beans by linking environmental conditions (e.g., temperature and relative humidity) to equilibrium moisture behavior. This enables operators to anticipate moisture uptake or loss, optimize ventilation and dehydration strategies, and reduce the risk of quality degradation, microbial growth, or physical damage during storage. Consequently, these digital models provide valuable decision-support tools for improving moisture management and overall storage efficiency at the industrial level.

## 4 Experimental Design, Materials and Methods

**Fig. 6:**
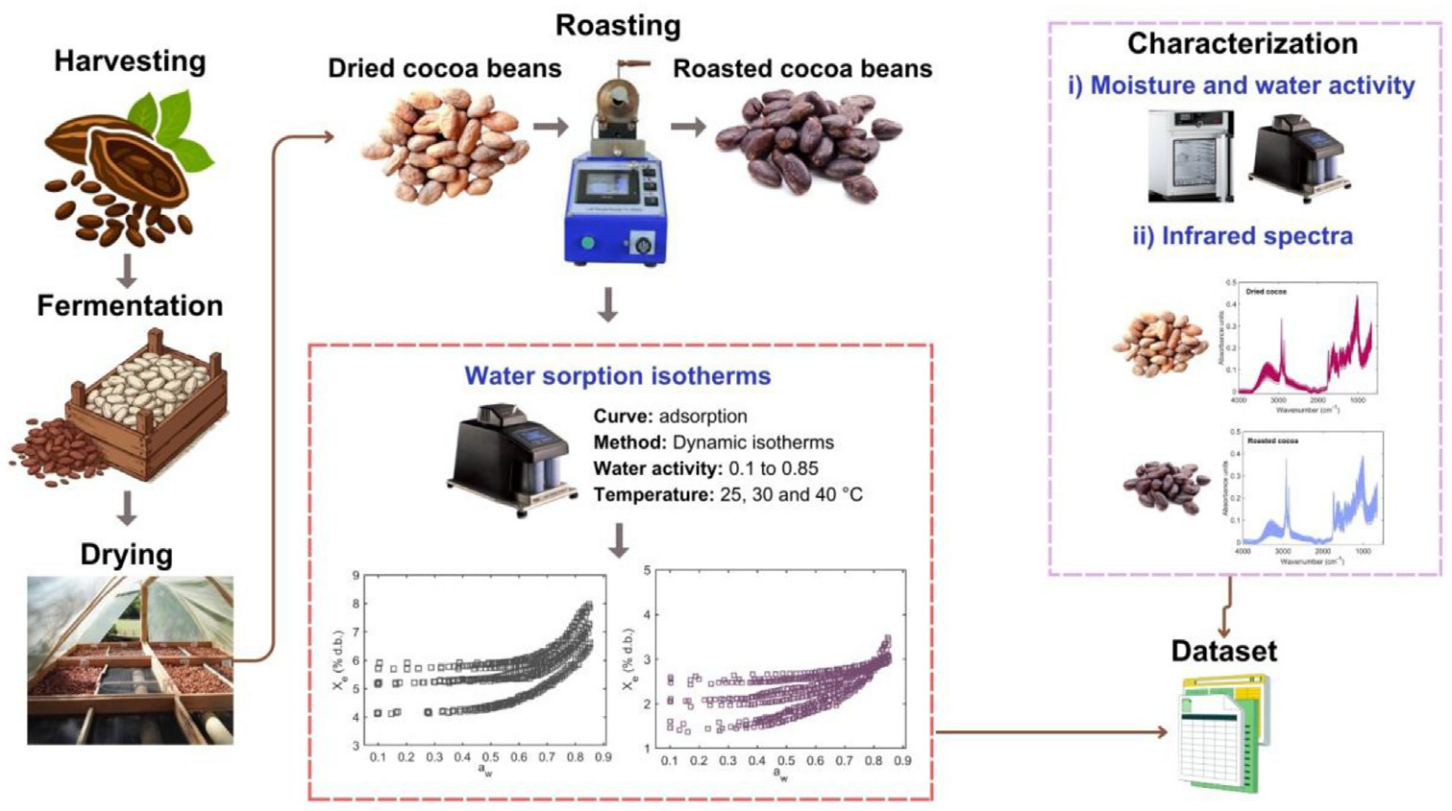
Schematic overview of the experimental workflow, including cocoa bean post-harvest processing, initial characterization (moisture content, water activity, and spectral properties), and determination of water adsorption isotherms for dried and roasted samples.

**Fig. 7:**
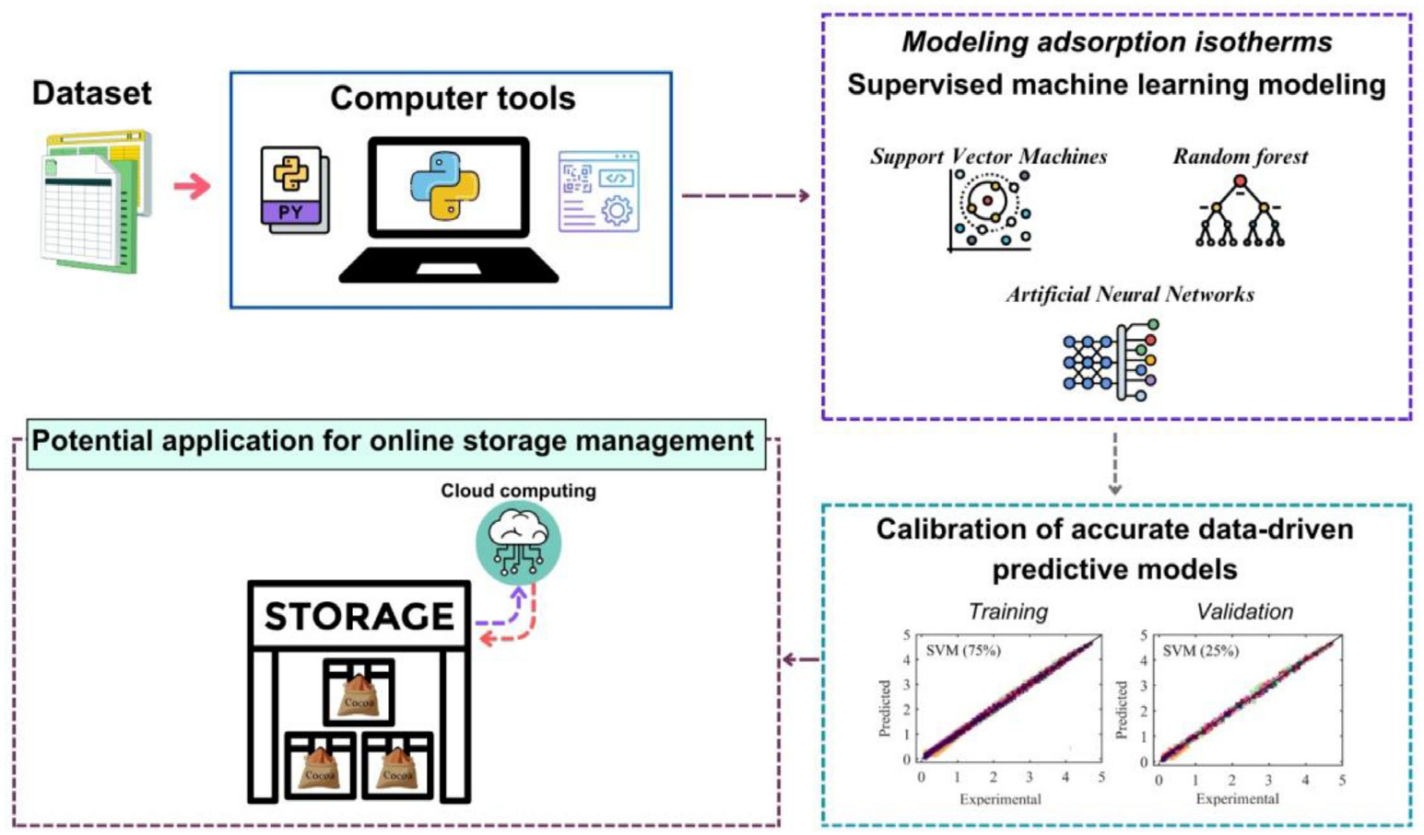
Flowchart of the computer-aided modeling procedure for applying machine learning techniques to calibrate predictive models on water adsorption isotherms of dried and roasted cocoa beans.

The experimental workflow employed for the processing of dried and roasted cocoa samples, the subsequent dataset processing, and python-based computational tools development are described in Fig. 6, Fig. 7. Fifteen cocoa bean samples (*Theobroma cacao* L.) were obtained from different cocoa farmers in the Huila region of Colombia and processed at the Centro Surcolombiano de Investigación en Café (CESURCAFÉ, Neiva-Huila, Colombia). All samples underwent standardized post-harvest processing (Fig. 6), including fermentation followed by sun drying under local environmental conditions [17]. Only healthy cocoa beans, free of visible defects, fungal contamination, or physical damage, were selected for subsequent analyses in order to ensure sample homogeneity and data reliability.

Roasting of cocoa beans was carried out at a laboratory scale using a rotary roasting system (TC-150 R, Quantik, Colombia) operated under controlled thermal conditions. Temperature profiles were continuously monitored throughout the roasting process to ensure process reproducibility and uniform heat transfer [18]. The roasting procedure followed a controlled thermal trajectory designed to simulate industrial cocoa roasting. Initially, the roasting chamber was preheated to 120 ± 2 °C, after which the dried cocoa beans were introduced into the system. The roasting process was maintained for a total duration of 20 ± 3 min, during which temperature fluctuations were kept within narrow limits (±2 °C) to ensure homogeneous roasting. After cooling to ambient temperature, the roasted cocoa beans were manually dehusked to remove the shell and obtain roasted cocoa nibs (Fig. 6). These nibs were subsequently used for initial characterization and experimental determination of water adsorption isotherms.

Moisture content of both dried and roasted cocoa samples was determined gravimetrically. Approximately 5 g of each sample was oven-dried at 105°C for 24 h until constant weight was achieved [19]. After drying, samples were cooled in a desiccator prior to weighing. The a_w_ of samples was determined using a vapor sorption analyzer (VSA Aqualab, Decagon Devices, Inc. Pullman, WA).

Mid-infrared spectra of dried and roasted cocoa samples was obtained using an FTIR spectrophotometer (Cary 630, Agilent, USA) coupled with a horizontal ATR sampling accessory (Diamond ATR) equipped with a ZnSe cell. ATR-FTIR spectra were acquired under controlled dry atmospheric conditions at room temperature (20 ± 0.5°C) [20]. Dried and roasted cocoa beans were individually ground using a rotating blade grinder and subsequently sieved. Approximately 1 g of each resulting cocoa powder was loaded onto the ATR sampling crystal and compressed to ensure adequate contact. Spectral data were collected over the wavenumber range of 4000–650 cm^–1^ at a resolution of 8 cm^–1^, averaging 20 scans per spectrum, followed by background correction. Each sample was measured in triplicate to ensure reproducibility.

Water adsorption isotherms of dried and roasted cocoa beans were experimentally determined using the DDI method. For each experiment, approximately 5 g of cocoa beans were placed inside a VSA (Aqualab, Decagon Devices Inc., Pullman, WA, USA). Prior to measurements, the instrument was calibrated according to the manufacturer’s specifications. Adsorption experiments were conducted over a water activity range of 0.10 to 0.85 at three temperatures representative of storage conditions (25, 30, and 40°C). The a_w_ resolution was set to 0.01, with an airflow rate of 100 mL min^–1^. All isotherms were performed in triplicate for each temperature and cocoa processing state (dried and roasted), resulting in a high-resolution dataset.

Data-driven mathematical modeling tools were developed using the Spyder IDE version 6.1.0, distributed through Anaconda Navigator and running on Python version 3.13.9. The computational framework was implemented in Python and relied on established scientific libraries, including *pandas* for data preprocessing and management, and *scikit-learn* for machine learning model development, training, validation, and performance assessment.

The developed Python scripts provide a structured, step-by-step workflow that enables users to apply ML techniques for the multivariate modeling of water adsorption isotherms of cocoa beans. Moreover, the proposed ML framework is inherently flexible and scalable, allowing for its extension to the mathematical modeling of water sorption phenomena in a wide range of agricultural and food products. These ML models can integrate additional process- and product-related variables such as formulation parameters, product variety, stage of processing, pretreatment conditions, and storage history, thereby enabling a comprehensive, high-dimensional description of complex food systems and significantly enhancing their predictive accuracy and decision-support capabilities.

The resulting ML-based predictive models are designed for subsequent deployment as digital decision-support tools in cocoa storage systems, facilitating robust moisture management and quality control. By employing statistically validated, data-driven models, this framework supports informed decision-making and lays the groundwork for the industrial implementation of intelligent systems aimed at optimizing storage operations and preserving cocoa bean quality.

## Limitations

None.

## Ethics Statement

The dataset acquired in this study did not involve human subjects, animal experiments, or data obtained from social media platforms.

## CRediT Author Statement

**Andrés F. Bahamón-Monje:** Software, Data curation, Writing, Original draft preparation. **Gentil A. Collazos-Escobar:** Conceptualization, Methodology, Software, Data curation, Visualization, Writing, Original draft preparation. **Nelson Gutiérrez-Guzmán:** Supervision, Writing- Reviewing and Editing.

## Data Availability

Mendeley DataDataset and Python-based Machine Learning tools for data-driven modeling of water adsorption isotherms in cocoa beans (Original data)
